# Electromagnetic Sensing and Its Applications

**DOI:** 10.3390/s26020574

**Published:** 2026-01-15

**Authors:** Wuliang Yin, Mingyang Lu, Ruochen Huang

**Affiliations:** 1 Department of Electrical and Electronic Engineering, University of Manchester, Manchester M60 1QD, UK; 2 Center for Nondestructive Evaluation, Iowa State University, Ames, IA 50011, USA; 3 College of Electrical Engineering and Automation, Fuzhou University, Fuzhou 350108, China; ruochen_huang@fzu.edu.cn

Electromagnetic sensing offers the ability to interact with the physical world beyond our five senses. The technique is used to interrogate the integrity and properties of materials in many industrial settings.

This Special Issue collected a small but diverse set of studies, covering applications ranging from measuring flow velocity [1], thickness [2,3], defects [4,5], steel microstructure [3], and magnetic fields [6,7] to evaluating shielding effectiveness [8], imaging of uniaxial objects [9] and generating leaky waves [10]. The reported frequency range spans from typical eddy current frequencies (below MHz) to radio frequency and up to microwave frequencies. The industrial sectors involved include the rail industry, the steel industry, etc., using multiphase flow measurements for magnetic measurement in synchrotrons. New techniques have been proposed such as data processing techniques using convolutional neural networks [9] and array antenna designs [10].

These issues show the versatility of the technique and the vibrant research activities carried out in our community.

Despite these achievements, important gaps remain. Many sensing techniques still face challenges in scaling from controlled laboratory environments to complex industrial settings, where noise, variability in material properties, and harsh operating conditions can limit accuracy and reliability. Furthermore, while individual applications have demonstrated success, there is a need for unified frameworks that connect electromagnetic sensing data with broader digital manufacturing and predictive maintenance systems. The field must also more deeply explore multiphysics interactions, especially in contexts such as multiphase flow measurement and fracture aperture monitoring, where electromagnetic signals must be interpreted alongside mechanical and fluid dynamics.

This Special Issue has contributed to bridging some of these gaps by presenting diverse approaches—from novel sensor designs to advanced data processing methods—that demonstrate both the versatility and adaptability of electromagnetic sensing. By showcasing applications across industries, the collected studies highlight how the community is actively pushing the boundaries of what can be measured and monitored.

Looking forward, several research directions stand out as promising:

- Integration with AI and machine learning: Beyond CNN-based imaging, future studies should explore adaptive algorithms capable of real-time decision-making in noisy industrial environments;
- Scalability and robustness: Developing sensors and systems that maintain accuracy under variable operating conditions remains a critical challenge;
- Multiphysics modeling: Coupling electromagnetic sensing with mechanical, thermal, and fluid models will enhance interpretation and predictive power;
- Miniaturization and portability: Compact, low-cost sensors could expand applications into new domains, including field inspections and consumer technologies;
- Standardization and interoperability: Establishing common protocols for data acquisition and interpretation will accelerate adoption across industries.

By addressing these areas, the community can ensure that electromagnetic sensing continues to evolve as a cornerstone technology for industrial diagnostics, materials characterization, and beyond. The progress documented here is a testament to vibrant research activity, and it sets the stage for the next wave of breakthroughs that will further extend our ability to interact with and understand the physical world.

Overall, we would like to thank all authors who have made great efforts to present their studies in their best way for this Special Issue, as well as the reviewers, all of whom have provided critical and constructive comments that contributed to the successful completion of this Special Issue.

Finally, I would like to dedicate this Special Issue to the late Professor Huaxiang Wang, who have devoted his life to the field of measurement and influenced many younger generations of researchers, including myself, as I benefited immensely from his mentorship during my MSc studies and later in my professional life.
